# Highly Efficient Phosphazene-Derivative-Based Flame Retardant with Comprehensive and Enhanced Fire Safety and Mechanical Performance for Polycarbonate

**DOI:** 10.3390/ma17133206

**Published:** 2024-07-01

**Authors:** Xiaowei Mu, Jing Zhan, Lu Liu, Zhongyi Yao, Yulu Zhu, Bin Yu, Lei Song

**Affiliations:** 1 State Key Laboratory of Fire Science, University of Science and Technology of China, Hefei 230026, China; xwmu@ustc.edu.cn (X.M.); leisong@ustc.edu.cn (L.S.); 2 School of Civil Engineering, Anhui Jianzhu University, Hefei 230601, China; 3 College of Materials Science and Engineering, Chongqing University, Chongqing 400044, China; 4 College of Materials Science and Engineering, Zhejiang University of Technology, Hangzhou 310014, China

**Keywords:** polycarbonate, flame retardancy, mechanical performance, pyrolysis mechanism

## Abstract

Polycarbonate (PC) as a widely used engineering plastic that shows disadvantages of flammability and large smoke production during combustion. Although many flame-retardant PCs have been developed, most of them show enhanced flame retardancy but poor smoke suppression or worsened mechanical performance. In this work, a novel nitrogen–phosphorus–sulfur synergistic flame retardant (Pc-FR) was synthesized and incorporated into PC with polytetrafluoroethylene (PTFE). The extremely low content of PC-FR (0.1–0.5 wt%) contributes significantly to the flame retardancy, smoke suppression and mechanical performance of PC. PC/0.3 wt% Pc-FR/0.3 wt% PTFE (PC-P0.3) shows the UL-94 V-0 and LOI of 33.5%. The PHRR, THR, PSPR, PCO and TCO of PC-P0.3 decreased by 39.44%, 14.38%, 17.45%, 54.75% and 30.61%, respectively. The impact strength and storage modulus of PC-P0.1 increased by 7.7 kJ/m^2^ and 26 MPa, respectively. The pyrolysis mechanism of PC-P0.3 is also revealed. The pyrolysis mechanism of PC-P0.3 is stochastic nucleation and subsequent growth and satisfies the Aevrami–Erofeev equation. The reaction order of PC-P0.3 is 1/2. The activation energy of PC-P0.3 is larger than PC-0, which proves that the Pc-FR can suppress the pyrolysis of the PC. This work offers a direction on how to design high-performance PC.

## 1. Introduction

Polycarbonate (PC), with excellent properties such as dimensional stability, transparency, good electrical insulation, high strength, impact resistance, fatigue resistance, wear resistance and aging resistance and that can withstand turning, milling, planning, grinding, drilling, stamping and other mechanical processing, has been widely used in various sectors of the national economy to replace non-ferrous metals such as copper, aluminum, zinc and non-metallic materials such as glass and wood [1,2]. PC has outstanding advantages in engineering applications, but it also has problems such as melt dripping and large smoke production during combustion. Therefore, in recent years, the flame-retardant and smoke-suppressing modification of PC has gradually become a hot topic [3]. Sulfonate chemicals, silicon chemicals and phosphorus chemicals are currently the most common commercial flame retardant systems [4,5,6].

Sulfonate flame retardants mainly have a gas-phase flame-retardant mechanism and can promote cross-linking and rearrangement of the PC in the condensed phase. Sulfonate flame retardants can achieve good flame retardant effects with the addition of very low amounts and can maintain the strength and transparency of PC, so they occupy a large proportion of the commercial market [7,8]. The most common sulfonate flame retardants mainly include potassium 3-phenylsulfonylbenzene sulfonate (KSS), sodium 2,4,5-trichlorobenzene sulfonate (STB) and potassium perfluoro butane sulfonate (PPFBS). Adding 0.05~0.1 wt% KSS makes the LOI of PC reach about 37% and has little impact on its transparency. In addition, KSS does not contain halogens, which has advantages in terms of environmental protection. Wang studied the thermal degradation process and thermal aging of PC with different KSS amounts using a thermogravimetric analyzer combined with pyrolysis kinetics fitting [9]. The addition of KSS reduces the activation energy of PC and accelerates weight loss of PC. Although it is beneficial to the thermal degradation of the PC, it also promotes the formation of char by PC in advance. However, sulfonate flame retardants cannot effectively inhibit heat release when PC burns.

Silicon-based flame retardants are mainly based on the condensed-phase char-forming mechanism [10,11]. Although the migration of silicon can enhance the surface strength of the carbon layer, it can also cause damage to the interior of the carbon layer. Silicone flame retardants are mainly divided into inorganic silicone flame retardants and organic silicone flame retardants. Common inorganic silicone flame retardants mainly include silica, silica/tin chloride, glass fiber, silicone gel/potassium carbonate, silicate/ammonium polyphosphate and hydrated silicon compound/ammonium polyphosphate. Although inorganic silicon compounds exhibit good flame retardant effects, they must be used in conjunction with other additives due to their poor compatibility with the polymer matrix and can easily affect the mechanical properties and processing properties of PC [12]. Organic silica flame retardant is a “green” flame retardant that has the characteristics of both inorganic compounds and organic polymers, with Si-O-Si as the main chain and organic groups as side chains. According to the type of substituent on the silicon atom, organic silica flame retardants can be divided into silicone resin (polysiloxane), methyl silicone resin, phenyl silicone resin, methylphenyl silicone resin, methoxy silicone resin and vinyl silicone, etc. However, the amount of organic silica flame retardant used in PC is significantly higher than that of sulfonate flame retardant [13,14].

Phosphorus flame retardants have both gas-phase and condensed-phase flame-retardant mechanisms. They capture free radicals and release inert gases while also increasing the char residue rate of the matrix [15,16,17,18,19,20]. Inorganic phosphorus flame retardants mainly include red phosphorus, black phosphorus, ammonium phosphate and phosphorus oxide, while organic phosphorus flame retardants include phosphate esters, organophosphates and phosphazenes. Among them, phosphazenes possess high thermal stability, a wide range of versatility in structure and properties and good compatibility with various polymers, allowing for easy incorporation into polymer formulations without significantly affecting the material’s mechanical or processing properties [21,22]. Triphenyl phosphate (TPP) has a flash point of only 220 °C and is not suitable for PC flame retardancy. Bis(diphenylphosphate) (RDP) and bisphenol A bis(diphenylphosphate) (BDP) have good flame retardant effects in both the gas phase and the condensed phase, but their flame retardant efficiency is lower than that of sulfonate flame retardants [23]. The 4.95 wt% hexaphenoxycyclotriphosphazene and 0.05 wt% PPFBS can effectively reduce the heat release and average effective combustion heat and can increase the ignition time of PC [24].

To sum up, organophosphorus flame retardants can increase char of polymer through the condensed-phase mechanism. Moreover, the phosphorus flame retardant itself will release a variety of gaseous phosphorus free radicals during the cracking process, which combine with the highly active hydroxyl radicals generated during the combustion, thereby blocking the chain reaction during combustion and achieving the purpose of meteorological flame retardancy [21,23]. The addition of N and S elements can play a synergistic effect with the phosphorus flame retardant in the condensed phase. On the one hand, it promotes the rearrangement and isomerization reaction of the PC chain. On the other hand, it increases the cross-linking degree and stability of the char layer [25,26]. Based on this synergistic mechanism, a new N-P-S synergistic flame retardant (Pc-FR) was synthesized using hexachlorocyclotriphosphazene and 5-amino-2-naphthalenesulfonic acid. The Pc-FR combines the characteristic of sulfonate flame retardants and phosphazenes. The structure versatility and good compatibility with various polymer matrices of Pc-FR contribute to the preparation of flame-retardant PC composite with a good comprehensive performance. It was compounded with polytetrafluoroethylene (PTFE) anti-drip agent in different proportions and then added to the PC matrix to prepare a series of Pc-FR/PC composite materials. The effect of Pc-FR on combustion, smoke suppression properties and mechanical properties of PC was studied. The influence of Pc-FR on PC pyrolysis and combustion has also been revealed through the study of the pyrolysis kinetics process of PC composite.

## 2. Materials and Methods

### 2.1. Materials

Polycarbonate (PC) (with a melt index of 10) was received from Bayer Ag (Leverkusen, Germany). Hexachlorotriphosphazene (HCCP) and 5-amino-2-naphthalene sulfonic acid were procured from Aladdin Chemical Reagent Co., Ltd. (Shanghai, China). Polytetrafluoroethylene anti-drip agent (PTFE) was purchased from Nanjing Tianshi New Material Technology Co., Ltd. (Nanjing, Jiangshu, China). Pyridine and NaOH were acquired from Sinopharm Chemical Reagent Co., Ltd. (Shanghai, China). The deionized water for washing was obtained from the laboratory.

### 2.2. Synthesis of N-P-S Synergistic Phosphorus Flame Retardant (Pc-FR)

**Figure 1:**
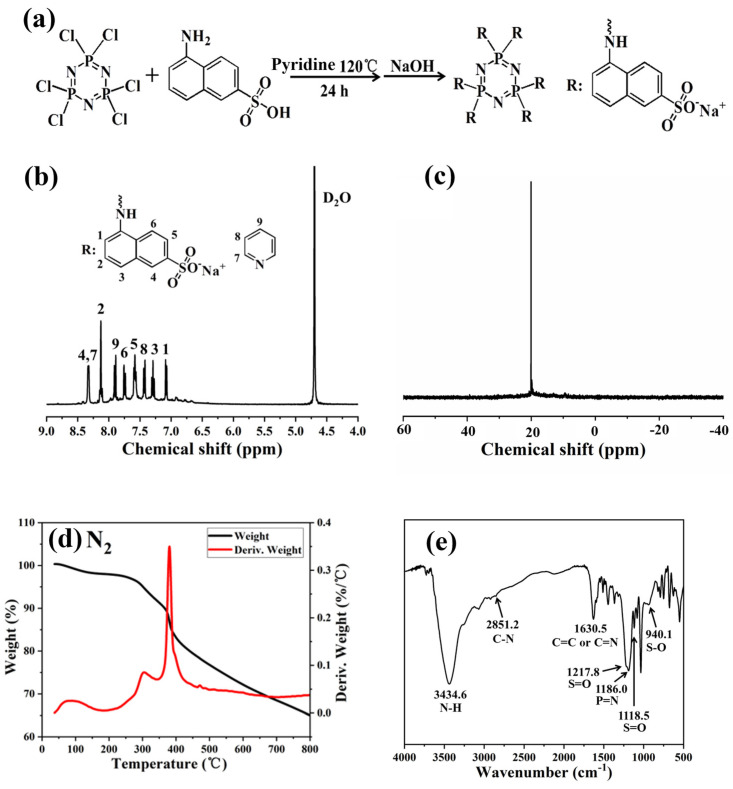
(**a**) Synthesis route of Pc-FR. (**b**) ^1^H and (**c**) ^31^P spectrum of Pc-FR. (**d**) TGA and DTG curves of Pc-FR in a nitrogen atmosphere. (**e**) FTIR curve of Pc-FR.

The Pc-FR was synthesized via the reaction as shown in Figure 1. First, 0.043 mol (9.60 g) of 5-amino-2-naphthalene sulfonic acid was dispersed in 100 mL of pyridine in a 250 mL single-mouth flask. After magnetic stirring for 2–3 h, a uniformly dispersed suspension was formed. Subsequently, 0.007 mol (2.43 g) of HCCP was dissolved in a small amount of pyridine solution and then was added dropwise to the above suspension at room temperature. At the same time, magnetic stirring was adjusted to a medium–high speed reaction for 1 h. Then, the flask was transferred to an oil pot at 120 °C for 24 h, and finally a large amount of purplish-black viscous solid was obtained. After removing most of the pyridine solvent by swirling evaporation, 200 mL of deionized water was added to dissolve the products and the insoluble impurities were removed by the filtration membrane extraction method. Finally, 0.043 mol (1.72 g) of NaOH was added to neutralize the sulfonate group into sodium sulfonate. After repeated rotation steaming and cleaning three times, the product was dried in an oven at 80 °C, and the final product was a purple-black solid and was ground into powder for use.

### 2.3. Preparation of Phosphorous Flame-Retardant PC Composites

PC composites with different contents of Pc-FR (0.1, 0.3, 0.5 wt%) were prepared by the melt blending method. The PC masterbatch was pre-dehydrated before use by heating in the oven at 80 °C for 72 h. The final PC composites were labeled as PC-PX, with X referring to the wt% of the synthesized Pc-FR. The specific method is as follows: The temperature in all regions of the mixer was set to 235 °C, about 50 g of pre-dehydrated pure PC masterbatch was added after the temperature became stable and the PC was melted completely into a viscous liquid at 80 rmp. Then, 0.15 g of PTFE (0.3 wt%) and the Pc-FR (0.05, 0.15 or 0.25 g) were added, and the composite was mixed for 10 min at 100 rpm to obtain the uniform composites. Finally, the samples were hot pressed by a plate vulcanizing machine at 235 °C for 10 min to obtain a 100 × 100 × 3 mm^3^ standard plate.

## 3. Results and Discussion

### 3.1. Characterization of Pc-FR

The chemical structure of the obtained flame retardant was characterized using nuclear magnetic resonance (NMR) and Fourier-transform infrared spectroscopy (FTIR). The ^1^H NMR and ^31^P NMR spectra of the synthesized Pc-FR are shown in Figure 1. From the ^1^H NMR (Figure 1b) spectrum, the chemical shift at 4.70 ppm is attributed to the deuterium of the solvent. Hydrogen in the amine is more active and can be easily replaced by deuterium water in exchange for heavy hydrogen, so there is no chemical shift of hydrogen in the amine in the spectrum. The main chemical shift peaks of the synthesized products are concentrated in the range of 7.0 to 8.3 ppm, in which 7.08 (1H, H1), 7.29 (1H, H3), 7.58 (1H, H5), 7.76 (1H, H6), 8.13 (1H, H2) and 8.32 (1H, H4) correspond to the characteristic peaks of hydrogen on branched naphthalene rings [27,28,29]. The chemical shift peak of sulfonic acid group hydrogen was not found near 2.0 ppm, indicating that all naphthalene sulfonic acid in the product was neutralized as sodium naphthalene sulfonic acid salt [30,31]. As shown in Figure 1c, the ^31^P NMR spectrum of the fabricated Pc-FR only shows one characteristic peak at around 20.1 ppm, confirming the existence of phosphazene in the synthetic N-P-S synergic phosphorous flame retardant, namely, all the six chlorine atoms on HCCP were replaced by aminobenzene sulfonic acid.

The fabricated flame retardant was further characterized by FTIR (Figure 1e). The characteristic peak centered at around 3434.6 cm^−1^ was ascribed to the N-H stretching on hydroxyl and naphthalene rings of water in the sample. The peak at 1630.5 cm^−1^ should be assigned to the bending vibration peak of the -NH group on the naphthalene ring, and the peaks at 2851.2 and 1368.2 cm^−1^ correspond to the C-N stretching vibration peak with double bond property in the amino naphthalene [32,33,34,35,36]. Meanwhile, combined with the analysis results of ^1^H NMR, the absorption peak at 1630.5 cm^−1^ is due to the stretching vibration peak of C=C and C=N double bonds in pyridine. The S-O stretching vibration peak of the sulfonate group corresponds to 940.1 cm^−1^, the S=O stretching vibration peaks relates to 1217.8 and 1118.5 cm^−1^ and the P=N characteristic peak of the phosphazene skeleton also can be found at 1186 cm^−1^ [37,38,39]. In comparison, the disappearance of the P-Cl bond at 601 and 530 cm^−1^ indicates that the original Cl atom in the structure of the product phosphazenes is completely replaced by aminonthalene sulfonate [40,41]. The elemental analysis is further applied to study the elemental composition of Pc-FR. The theoretical and practical elements content of Pc-FR are listed in Table 1. The practical C, H, N, S and P of Pc-FR are 36.14%, 3.62%, 6.22%, 8.89% and 5.84%, respectively.

To verify whether the thermal stability of the Pc-FR can satisfy the application and processing requirements of PC, thermogravimetric tests of Pc-FR were carried out under nitrogen and air conditions. As shown in Figure 1d, in a nitrogen atmosphere, the mass loss of Pc-FR is only slight in the temperature range from 30 to 140 °C, suggesting that the flame retardants lost water and residual pyridine solvent when being heated. The weight loss of the Pc-FR can be divided into two stages from 250 to 390 °C, the mass decreases from 97.36% to 84.42% and the corresponding temperature at the maximum thermal weight loss rate (T_m_) is 381.1 °C. The mass of the sample is still 65.06% at 800 °C. Concurrently, The Pc-FR also has only a small mass loss at 30 to 140 °C in the air atmosphere (Figure S1). However, there are five weight loss steps in the range of 250 to 800 °C, with the mass decreasing from 96.93% to 26.11% and the T_m_ of 369.2 °C. The initial decomposition temperature of the fabricated flame retardant is higher than the processing temperature of PC (235 °C). Hence, the thermal stability of the synthetic flame retardant can meet the requirement of PC utilization and manufacturing.

### 3.2. Flammability of PC and Its Composite

The flame retardancy of PC and its composite were primarily evaluated by using a UL-94 vertical burning tester and limiting oxygen index (LOI), and the detailed data are listed in Table 2. As shown in Figure S2, upon ignition, the pure PC melts rapidly, followed by evident melt-dripping. Although the melt dripping can take away the heat released by the combustion of the PC, the burning droplets ignite the absorbent cotton below the spline; therefore, there is no rating of vertical combustion of the pure PC. The melt-dripping phenomenon of the sample during combustion disappears conspicuously after the addition of a small amount (0.3 wt%) of anti-drip agent of PTFE, for which t_1_ is only 8.28 s, but t_2_ is 47.28 s, so the PC/PTFE achieves a UL-94 V-2 rating. When 0.1 wt% of the Pc-FR (PC-P0.1) is incorporated, both t_1_ and t_2_ decrease significantly, but the total extinguishment time is more than 10 s; thus, the sample achieves a V-1 rating. However, PC-P0.3 reached V-0 at 0.3 wt% loading of Pc-FR. Moreover, the PC composite with 0.5 wt% of Pc-FR can achieve a UL-94 V-0 rating. Simultaneously, with the increase in the addition of the Pc-FR, t_1_ slightly increases, while t_2_ significantly abates, which is due to the mechanism of Pc-FR promoting the pyrolysis of PC into carbon at the initial stage of combustion. In terms of the LOI, PTFE can increase the LOI of PC to 30%, while 0.1 wt% Pc-FR and 0.3 wt% PTFE can increase the LOI value of PC to 34.5% sequentially. Notably, the LOI value of the PC composite decreases slightly with the extra increase in Pc-FR, and it also can remain stable at 33.5%.

### 3.3. Combustion Behaviors of PC and Its Composite

**Figure 2:**
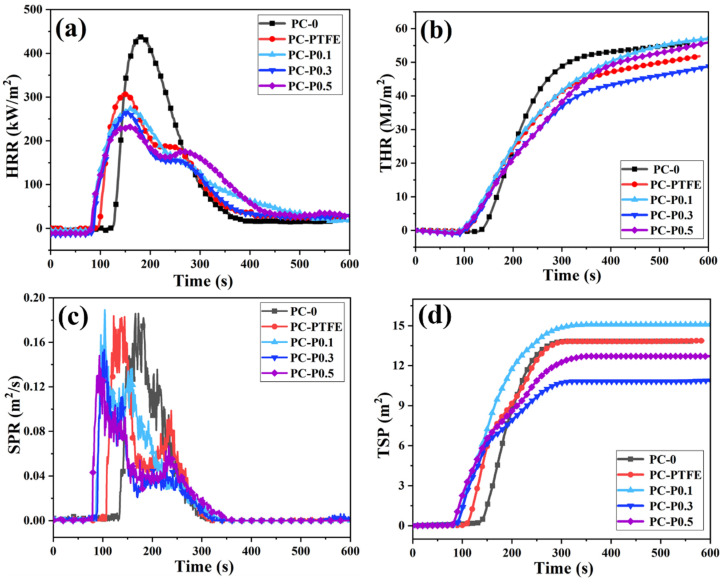
(**a**) HRR, (**b**) THR, (**c**) SPR and (**d**) TSP curves of PC and its composites.

To further explore the effects of the addition amount of Pc-FR on fire hazards such as heat release, smoke release and smoke toxicity during PC combustion, cone calorimetry tests were conducted, and detailed data are listed in Table 3. The heat release rate (HRR) and total heat release (THR) curves of PC and its composite are shown in Figure 2. The peak heat release rate (PHRR) of samples reduces from 440.61 kW/m^2^ to 307.90 kW/m^2^ when only 0.3 wt% anti-drip PTFE is added, decreasing by 30.12%. However, the ignition time (TTI) of PC-PTFE is 92 s, 31 s earlier than that of pure PC. After the addition of Pc-FR, the PHRRs of the PC-P0.1, PC-P0.3 and PC-P0.5 decrease to 273.74 kW/m^2^, 266.82 kW/m^2^ and 235.80 kW/m^2^ by 37.87%, 39.44% and 46.48%, respectively, while the TTI value also decreased to about 76 s. In addition, the HRR curves of the PC composites show two heat release peaks, indicating that the carbon layer generated in the first stage is partially destroyed, and the combustible gas escapes during the continuous heating process. The above phenomena are closely linked to the fire-retardant mechanism of phosphorous-based flame retardants, which further release flame-retardant gases and capture free radicals. Nevertheless, they have an adverse impact on the structure of the carbon layer, resulting in the emergence of a secondary heat release peak.

Owing to the HRR peak of PC being widened by the addition of anti-drip PTFE and Pc-FR, the total heat release (THR) should be analyzed. The THR of PC decreases from 55.63 to 51.31 MJ/m^2^ with the addition of PTFE. The THR of the PC-P0.3 is only 47.63 MJ/m^2^, which decreases by 14.38%. In contrast, the THR of the PC-P0.1 and PC-P0.5 is 56.37 and 54.69 MJ/m^2^, respectively, and even increases compared with the pure PC. This may be ascribed to Pc-FR being mainly a gas-phase flame retardant, and an insufficient or excessive amount of Pc-FR is inconducive to the formation of high-quality residual char. As shown in Table 3, the amount of char residue of the PC composites is lower than that of the PC-PTFE. The amount of char residue of PC-P0.1 and PC-P0.5 is even similar to that of the pure sample, which also supports the above analysis.

To compare the influence of Pc-FR on the smoke release of PC during combustion, the smoke production rate (SPR) and total smoke production (TSP) are shown in Figure 2. It is interesting to find that all the samples except pure PC have two smoke release peaks, which may be assigned to the destruction and decomposition of the char residue. The addition of PTFE does not curtail the peak smoke release rate (PSPR) or TSP of PC and conversely even accelerates the smoke release. The PSPR and TSP of the PC-P0.3 are reduced by 17.45% and to 12.71 m^2^, respectively. Although the TSP of PC-P0.5 is inhibited, the smoke suppression effect is inferior to that of the PC-P0.3. The addition of 0.1 wt% Pc-FR even exacerbates the smoke suppression performance of PC, with an increased TSP of 15.08 m^2^.

**Figure 3:**
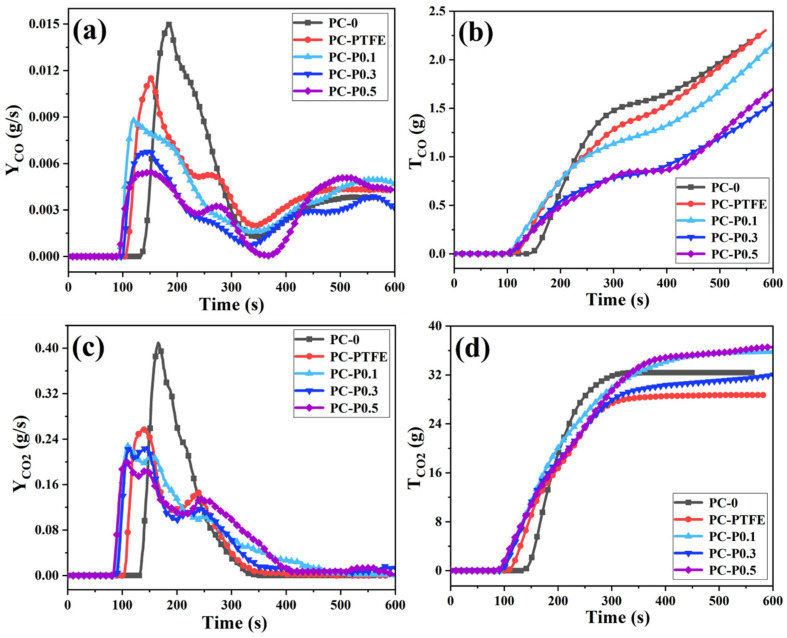
(**a**) Yco, (**b**) Tco, (**c**) Yco_2_ and (**d**) Tco_2_ curves of PC and its composites.

The toxic gases produced by polymer materials in the combustion process are mainly CO and CO_2_. Therefore, the release rate (Y_CO_, Y_CO2_) and total release amount (T_CO_, T_CO2_) of the two gases were collected, and their changing trends are shown in Figure 3. Although the initial release time of CO is advanced, as the amount of PC-FR added increases (0.1 wt%, 0.3 wt% and 0.5 wt%), its peak release rate (PCO) gradually decreases, (deceasing by 41.30%, 54.75% and 63.65%). The T_CO_ of PC-P0.3 is 1.406 g/s, which is 30.61% lower than that of pure sample. In addition to CO release, the addition of PC-FR is found to considerably reduce the CO_2_ production of PC during burning. The peak CO_2_ release rate (P_CO2_) is inhibited, and it appears a double peak, while the T_CO2_ of all the samples with Pc-FR is significantly higher than that of PC-PTFE and even exceeds the pure PC. This phenomenon may be attributed to the decomposition of the char layer caused by Pc-FR.

### 3.4. Pyrolysis Behavior and Mechanism of PC and Its Composites

#### 3.4.1. Effect of Heating Rate on Weight Loss of PC and Its Composite

**Figure 4:**
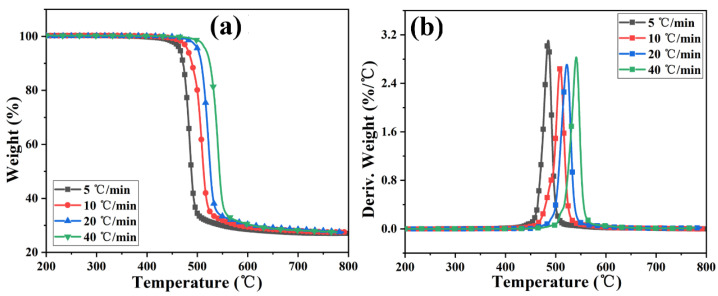
(**a**) TGA and (**b**) DTG curves of PC-P0.3 at different heating rates.

**Figure 5:**
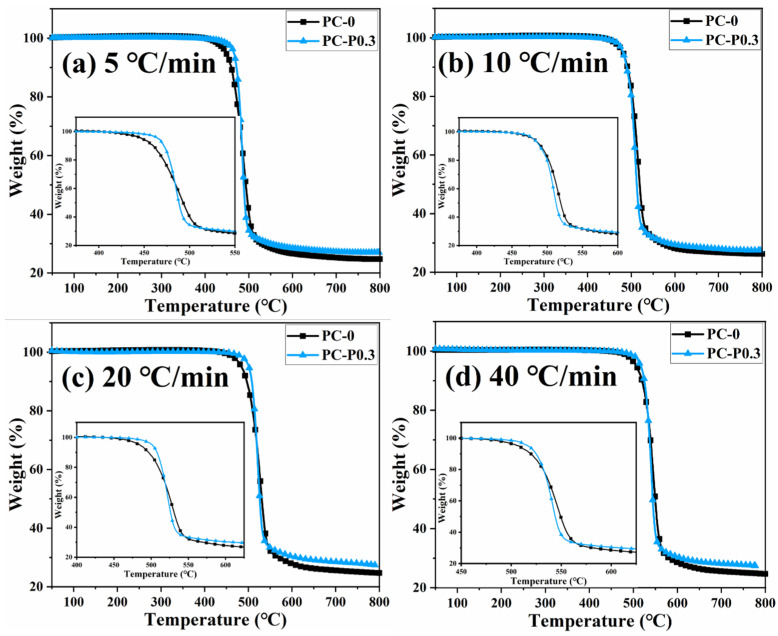
TGA curves of PC and PC-P0.3 at heating rates of (**a**) 5 °C/min, (**b**) 10 °C/min, (**c**) 20 °C/min and (**d**) 40 °C/min.

**Figure 6:**
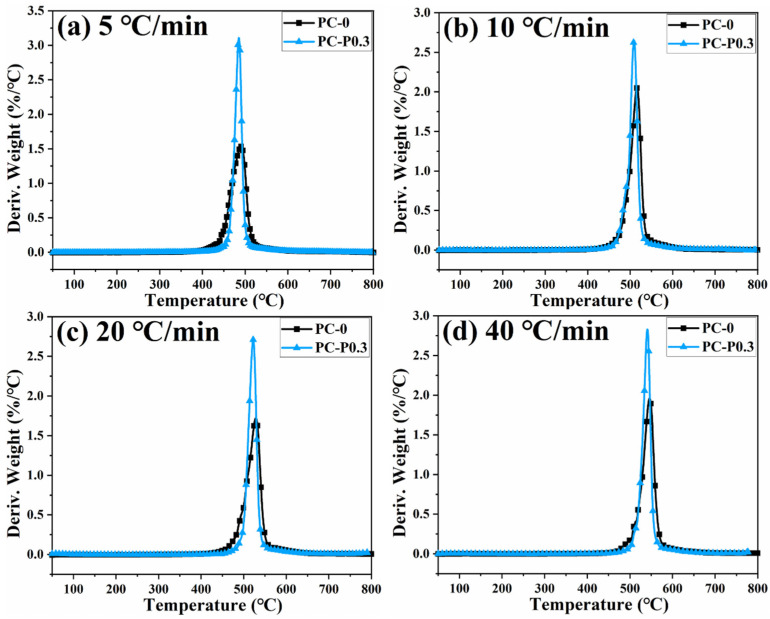
DTG curves of PC and PC-P0.3 at heating rates of (**a**) 5 °C/min, (**b**) 10 °C/min, (**c**) 20 °C/min and (**d**) 40 °C/min.

Figure 4 and Figure S3 show the TGA and DTG curves of PC and its composite at different heating rates. The TGA curve of the sample moves toward a high temperature with the increased heating rate. The initial decomposition temperature and the temperature at the highest weight loss rate (T_p_) of the sample increase, while the amount of char residue does not change significantly. The TGA curves of PC and its composites at different heating rates are shown in Figure 5. The T_5wt%_ and T_10wt%_ of the PC composite are significantly higher than those of the pure PC (increasing by about 10 °C compared with the pure sample at each heating rate). In addition, the amount of pyrolysis char residue of PC-P0.3 increases by approximately 11% compared with pure PC. In a nitrogen atmosphere, Pc-FR increases the initial decomposition temperature of PC and promotes char formation, thereby improving the thermal stability of PC. The DTG curves of PC and its composite are shown in Figure 6. As shown in Table 4, The T_50wt%_ and T_p_ of the PC-P0.3 are about 5 °C lower than those of pure PC. This may be due to the excellent gas-phase flame retardant effect of the Pc-FR, which causes the maximum pyrolysis rate to appear earlier. The PC with Pc-FR has the highest peak DTG value and can be quickly decomposed, which is consistent with its gas-phase flame retardant mechanism.

#### 3.4.2. Activation Energy Calculation

**Figure 7:**
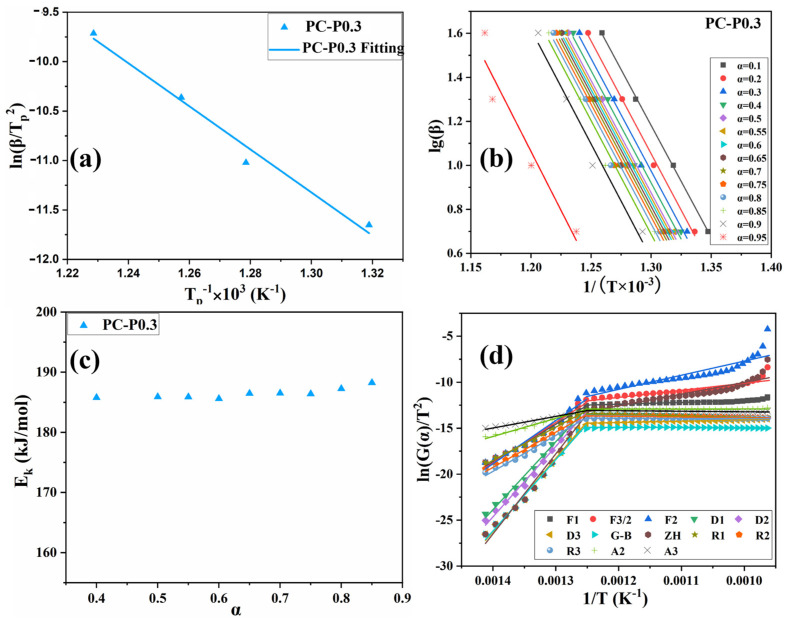
(**a**) Fitting curve of PC-P0.3 based on Kissinger method. (**b**) Fitting curve of PC-P0.3 based on F-W-O method. (**c**) Activation energy of PC-P0.3 at different conversion rates in F-W-O method. (**d**) Fitting curve of PC-P0.3 based on DAEM method.

**Figure 8:**
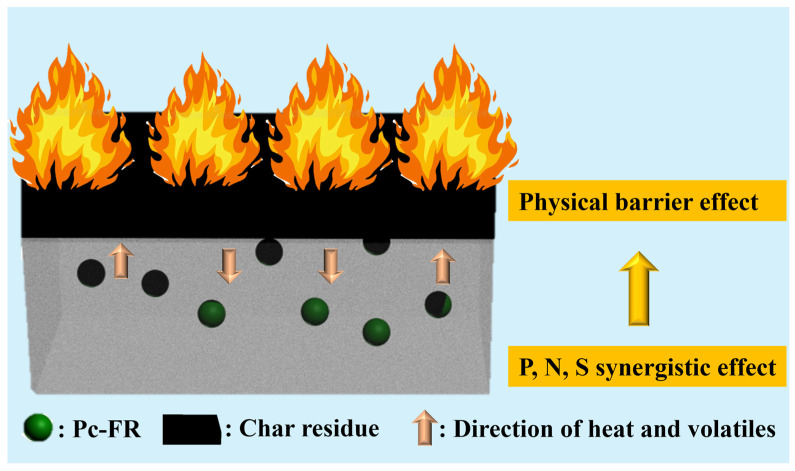
Flame retardant mechanism of Pc-FR.

In Figure 7a and Figure S4a and Table S1, the activation energy obtained by the Kissinger method is PC-0 > PC-P0.3. The E_k_ of both PC-0 and PC-P0.3 is around 181 kJ/mol, proving that Pc-FR has no significant effect on the pyrolysis reaction of the PC. The F-W-O method fitting curves of PC and PC-P0.3 are shown in Figure 7b and Figure S4b. When α was set to 0.1, 0.2, 0.3, 0.4, 0.5, 0.55, 0.6, 0.65, 0.7, 0.75, 0.8, 0.85, 0.9 and 0.95, the corresponding T values of the PC with different β values were taken, and linear fitting was carried out. The relationship between the E_k_ value and α is shown in Figure 7c and Figure S4c. Relevant data are listed in Table S2. The increase in conversion rate has little effect on the activation energy of the pyrolysis reaction of the PC-P0.3, and its E_k_ value only fluctuates around 186.45 kJ/mol. Combined with the analysis of the flame retardant mechanism, it can be seen that although the dominant flame retardant role of Pc-FR changes from the gas phase to the condensed phase, the two flame retardant mechanisms are always relatively coordinated during this process, and the effect of inhibiting the thermal decomposition of PC is stable. Therefore, the pyrolysis activation energy of the sample can still maintain at a high level at different conversion rates. Selecting G (α) in Table S3 one by one, performing mathematical processing on the thermogravimetric data of PC and PC-P0.3 (β is 10 °C/min) and making ln[G (α)/T^2] pair and performing data fitting. The results are shown in Figure 7d and Figure S4d. The correlation coefficient R’ values obtained by fitting PC and PC-P0.3 according to different pyrolysis mechanism functions G (α) are recorded in Table S4. The closer the R’ value is to 1, the better the fitting effect is. The pyrolysis mechanism of PC and its composite is stochastic nucleation and subsequent growth, and both meet the Aevrami–Erofeev (A2) equation, and the reaction order is 1/2. The activation energy of the sample is: PC-P0.3 > PC-0. Pc-FR can increase the E_k_ of the PC by 12.8 kJ/mol, significantly enhancing the thermal stability of the PC matrix. The detailed flame-retardant mechanism of Pc-FR is shown in Figure 8. The Pc-FR combines the characteristic of sulfonate flame retardants and phosphazenes. The N and S elements can play a synergistic effect with the P element of Pc-FR in the condensed phase, resulting in an enhanced physical barrier effect of char residue. Thus, the flame retardancy of PC is enhanced.

### 3.5. Mechanical Performances of PC and Its Composites

**Figure 9:**
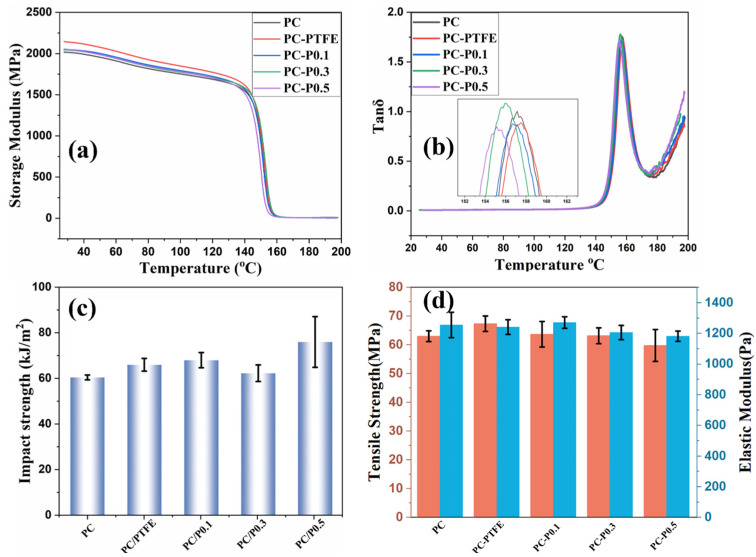
(**a**) Storage modulus, (**b**) tanδ, (**c**) impact strength and (**d**) tensile strength and elastic modulus of PC and its composites.

In addition to the fire risk of the PC composite, the mechanical performance of PC and its composites were studied. As shown in Figure 9a,b and Table S5, the storage modulus of PC improved slightly after incorporating the Pc-FR and PTFE. The storage modulus of PC increases from 2015 MPa to 2046 MPa after the addition of 0.3 wt% of Pc-FR. However, the glass transition temperature (Tg) of PC decreases slightly after the addition of Pc-FR. The Tg of PC-P0.3 is 1.3 °C lower than that of PC. Apart from the enhanced storage modulus, the incorporation of PC-FR and PTFE also contributes to the impact strength and tensile strength of PC. The impact strength of PC increases from 61.1 kJ/m^2^ to 62.9 kJ/m^2^ after the addition of 0.3 wt% Pc-FR. The tensile strength of PC increases from 24.48 MPa to 30.49 MPa after the addition of 0.3 wt% Pc-FR.

## 4. Conclusions

In this work, a new N-P-S synergistic flame retardant (Pc-FR) was successfully synthesized by completely replacing the chlorine atoms in hexachlorocyclotriphosphazene with aminonaphthalene sulfonic acid, and flame-retardant PC composite with different addition ratios was prepared. When adding 0.3 wt% of Pc-FR to PC, the PC reaches the UL-94 V-0 level, and the LOI increases from 25.5% to 33.5%. The 0.3 wt% Pc-FR and 0.3 wt% PTFE can reduce the PHRR, THR, PSPR, P_CO_ and T_CO_ of PC by 39.44%, 14.38%, 17.45%, 54.75% and 30.61%, respectively. In addition to enhanced fire safety, the PC-FR contributes to the mechanical performance of PC. The storage modulus, impact strength and tensile strength of PC are also improved. The impact strength of PC increases from 61.1 kJ/m^2^ to 62.9 kJ/m^2^ after the addition of 0.3 wt% Pc-FR. The tensile strength of PC increases from 24.48 MPa to 30.49 MPa after the addition of 0.3 wt% Pc-FR.

The pyrolysis behavior and mechanism of PC and its composites were also investigated. By studying the pyrolysis characteristics of PC and its composite at different heating rates, it is found that Pc-FR can increase the initial decomposition temperature and char residue amount of the PC during pyrolysis. It can be seen from the Kissinger method that the activation energy of the PC-P0.3 is approximately 186 kJ/mol, which is almost the same as that of pure PC. In addition, the calculation results of the F-W-O method can prove that the activation energy of PC-P0.3 always remains at a high level, indicating that the conversion rate has little impact on its pyrolysis reaction process. After further calculation and comparison of the pyrolysis mechanism functions of PC and its composite, it is found that the pyrolysis mechanism of PC-P0.3 is stochastic nucleation, and subsequent growth and satisfies the Aevrami–Erofeev (A2) equation. The reaction order of PC-P0.3 is 1/2. The activation energy of the samples from large to small is PC-P0.3 > PC-0. It is proved that the Pc-FR can indeed play a certain role in inhibiting the thermal decomposition of the PC matrix. This work provides a guideline for how to design PC composite with high fire safety and enhanced mechanical performance.

The Pc-FR combines the characteristic of sulfonate flame retardants and phosphazenes. The structure versatility and good compatibility with various polymer matrices of Pc-FR contribute to the preparation of flame-retardant PC composite with a good comprehensive performance. The N and S elements can play a synergistic effect with the P element of Pc-FR in the condensed phase. Thus, the Pc-FR shows a high flame-retardant efficiency compared with P- or Si-based flame retardant. However, the cost of Pc-FR is significantly higher than commercial PC flame retardant. The amount of Pc-FR added to make PC reach V0 is also larger than that of commercial sulfonate PC flame retardant.

## Figures and Tables

**Table 1:** Element content of Pc-FR.

Element Content	C	N	H	P	S
Theoretical content	44.04%	7.71%	2.57%	5.69%	11.74%
Practical content	36.14%	6.22%	3.62%	5.84%	8.89%

**Table 2:** UL-94 and LOI of PC and its composites.

Samples	Formulation (wt%)			UL-94			LOI (%)
	PC	PTFE	Pc-FR	t_1_ (s)	t_2_ (s)	Rating	
PC-0	100	-	-	dripping and then extinguishing		NR	25.5
PC-PTFE	99.7	0.3	-	8.28	47.28	V-2	30
PC-P0.1	99.6	0.3	0.1	2.04	11.12	V-1	34.5
PC-P0.3	99.4	0.3	0.3	2.56	4.37	V-0	33.5
PC-P0.5	99.2	0.3	0.5	2.94	1.09	V-0	33.5

t_1_ and t_2_ are flame duration after the first and second ignition, respectively. NR: No rating.

**Table 3:** Cone calorimetry data of PC and its composites.

Samples	PC-0	PC-PTFE	PC-P0.1	PC-P0.3	PC-P0.5
TTI (s)	123	92	77	76	73
PHRR (kW/m^2^)	440.61	307.90	273.74	266.82	235.80
THR (MJ/m^2^)	55.63	51.31	56.37	47.63	54.69
Char residues (%)	24.48	33.49	26.42	30.49	24.21
PSPR (m^2^/s)	0.1857	0.1838	0.1892	0.1533	0.1479
TSP (m^2^)	13.83	13.86	15.08	10.83	12.71
P_CO_ (g/s)	0.01494	0.01150	0.00877	0.00676	0.00543
P_CO2_ (g/s)	0.4096	0.2596	0.2292	0.2260	0.2023
T_CO_ (g)	2.205	2.196	1.967	1.406	1.530
T_CO2_ (g)	32.37	28.71	35.73	31.53	36.31

**Table 4:** TGA of PC and its composite at different heating rates.

Sample	β (°C/min)	T_5%_ (°C)	T_10%_ (°C)	T_50%_ (°C)	Char (%)	T_p_ (°C)	P_DTG_ (%/°C)
PC-0	5	447.65	459.72	492.96	24.77	490.19	1.5336
	10	480.27	490.89	519.09	25.23	515.94	2.0507
	20	484.60	496.39	530.60	24.70	528.44	1.7218
	40	507.80	519.43	549.33	24.70	546.34	1.9587
PC-P0.3	5	465.33	471.51	488.19	27.05	485.07	3.1080
	10	480.80	489.28	512.42	27.52	509.00	2.6509
	20	501.36	507.76	525.92	27.45	522.19	2.7095
	40	516.67	524.74	543.73	27.44	540.81	2.8298

## Data Availability

The original contributions presented in the study are included in the article and Supplementary Materials, further inquiries can be directed to the corresponding authors.

## Supplementary Materials

The following supporting information can be downloaded at: https://www.mdpi.com/article/10.3390/ma17133206/s1, Characterization; Activation energy calculation; Figure S1: TGA and DTG curves of Pc-FR in an air atmosphere; Figure S2: Digital images of PC and its flame-retardant composite; Figure S3: (a) TGA and (b) DTG curves of PC at different heating rates; Figure S4: (a) Fitting curve of PC based on Kissinger method. (b) Fitting curve of PC based on F-W-O method. (c) Activation energy of PC at different conversion rates in F-W-O method. (d) Fitting curve of PC based on DAEM method; Table S1: Fitting data of PC and its composite through Kissinger method; Table S2: Fitting data of PC and its composite through F-W-O method; Table S3: Common kinetic models and typical mechanism functions; Table S4: Correlation coefficient R’ value obtained after fitting different pyrolysis mechanism functions of PC and its composites; Table S5: Mechanical performances of PC and its composites.
