# Supplementary material for: Highly Efficient Phosphazene-Derivative-Based Flame Retardant with Comprehensive and Enhanced Fire Safety and Mechanical Performance for Polycarbonate
Source: Materials (Basel). 2024 Jul 1;17(13):3206. doi: 10.3390/ma17133206 (PMC11242893; doi:10.3390/ma17133206)
Supplement: Supplementary file 1 [file materials-17-03206-s001.zip › materials-3049046-supplementary.pdf]

Highly efficient phosphazene-derivative-based flame retardant with comprehensive and enhanced fire safety and mechanical performance for polycarbonate

Xiaowei Mu [1], Jing Zhan [2,*], Lu Liu [3], Zhongyi Yao [4], Yulu Zhu [4], Bin Yu [1,*] and Lei Song [1]

[1] State Key Laboratory of Fire Science, University of Science and Technology of China, Hefei 230026, China

[2] School of Civil Engineering, Anhui Jianzhu University, Hefei 230601, China

[3] College of Materials Science and Engineering, Chongqing University, Chongqing 400044, China

[4] College of Materials Science and Engineering, Zhejiang University of Technology, Hanzhou 310014, China

* Correspondence: zhanjing@ahjzu.edu.cn (J.Z.); yubin@ustc.edu.cn (B.Y.); Tel./Fax: +86-551-6360-2353 (B.Y.)

1 Experiment

1.1 Characterization

^{1}H NMR and ^{31}P NMR of the Pc-FR were measured on a Bruker AV400 instrument (Germany), and deuterium water was selected as the solvent.

A Nicolet 6700 FTIR spectrophotometer (USA) was used to characterize the chemical structure of synthetic Pc-FR, and the wavenumber range was set from 4000 to 500 cm^{-1}.

The thermogravimetric analysis (TGA) was carried out to characterize the thermal stability of synthetic samples using a TGA Q5000 IR thermogravimetric analyzer (USA) with a heating rate of 20°C/min in nitrogen and air atmosphere.

UL-94 vertical burning tests were performed according to ASTM D3801-1996, and a vertical burning tester (CZF-Ⅱ, Nanjing Jiangning Analytical Instrument Factory, China) was used in this test.

The LOI test was carried out according to ASTM D2863-2010. The apparatus used for this test was an HC-2 oxygen index meter (Jiangning Analysis Instrument Company, China).

Combustion behavior was measured in a cone calorimeter (Ttech-GBT16172, Ttech (Suzhou) Testing Instrument Technology Co., LTD., China). The heat release, smoke production and smoke toxicity of the PC composites with a dimension of 100×100×3 mm^3 were tested in the combustion process, and samples were irradiated with a heat flux of 35 kW/m^2.

The tensile strength of samples was assessed on a WD-20D electronic universal testing instrument (Changchun Intelligent Instrument Co., Ltd., China) according to the Chinese standard method (GB 13022–91). Each sample was tested three times.

Dynamic mechanical analysis (DMA) was analyzed by a DMA Q800 instrument (TA Instruments Inc., USA) at a fixed frequency of 1 Hz.

1.2 Activation energy calculation

1.2.1 Kissinger method to calculate activation energy

The T_p of the sample at different heating rates is used as the basic data in Kissinger method, and the activation energy of the material is obtained by solving the slope of the linear fitting equation. It is one of the most common pyrolysis kinetic differential analysis methods. Its calculation formula is shown in equation (1)

$$\ln\left(\frac{\beta}{T_p^2}\right) = \ln\left(\frac{AR}{E}\right) - \frac{E_k}{RT_p} \qquad (1)$$

where β is the heating rate; Tp is the peak pyrolysis rate; A is the pre-factor; R is the ideal gas constant and E_k is the activation energy of the material.

1.2.2 F-W-O method to calculate activation energy

The same mass conversion rate at different heating rates is selected using the F-W-O method so that the integral form of the kinetic mechanism function G(α) becomes a constant value. This method avoids the choice of reaction mechanism function and avoids errors caused by different mechanism function assumptions. The calculation formula is shown in equation (2).

$$\lg(\beta) = \lg\left(\frac{AR}{RG(\alpha)}\right) - 2.315 - 0.4567\frac{E_k}{RT} \qquad (2)$$

where β is the heating rate; A is the former factor; R is the ideal gas constant; G(α) is the kinetic mechanism function in integral form; α is the mass conversion rate; E_k is the activation energy of the material and T is the corresponding temperature at the same conversion rate.

1.2.3 Most probable mechanism function

When describing the kinetic problems involved in reaction equation (10), there can be two different forms of equations as shown in (4) and (6).

$$A'(s) \longrightarrow B'(s) + C'(s) \tag{3}$$

$$\frac{d\alpha}{dt} = kf(\alpha) \tag{4}$$

$$G(\alpha) = kt \tag{5}$$

where α is the fraction of substance A' that has been reacted at time t; t is the reaction time; k is the reaction rate constant; f(α) is the kinetic mechanism function in differential form and G(α) is the kinetic mechanism function in integral form.

In the non-isothermal kinetic analysis of materials, large errors are often caused due to the difference between the selected G (α) or f (α) form and the actual kinetic process. Therefore, by introducing the highly reliable Coats–Redfern integral formula (6), the material can be further processed dynamically.

$$\ln\left(\frac{G(\alpha)}{T^2}\right) = \ln\left(\frac{AR}{\beta E_k}\right) - \frac{E_k}{RT} \tag{6}$$

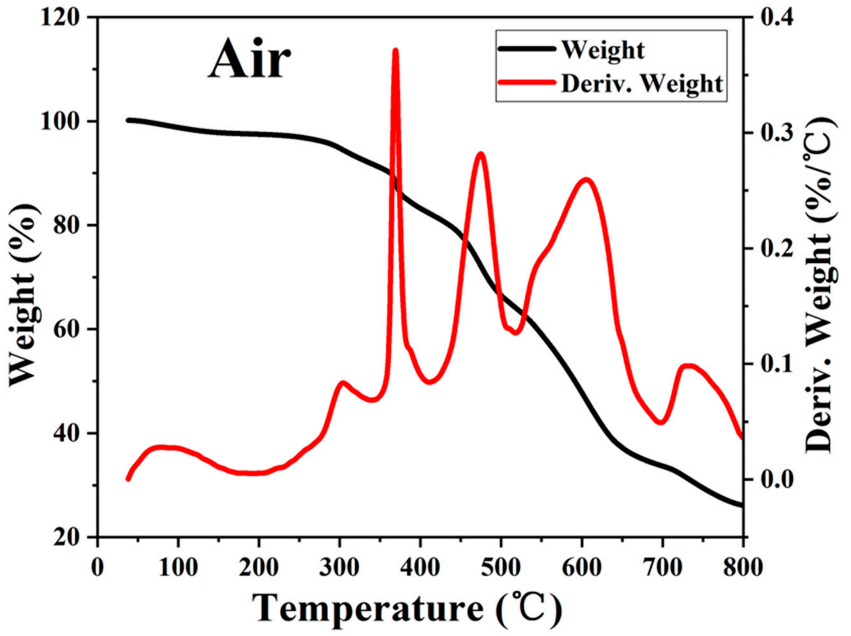

Figure S1 TGA and DTG curves of Pc-FR in an air atmosphere.

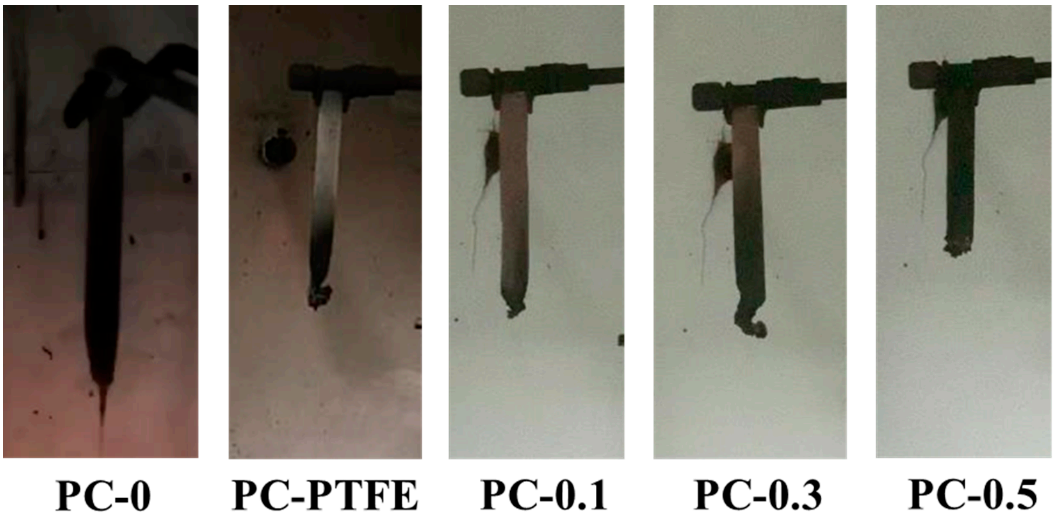

Figure S2 Digital images of PC and its flame-retardant composite.

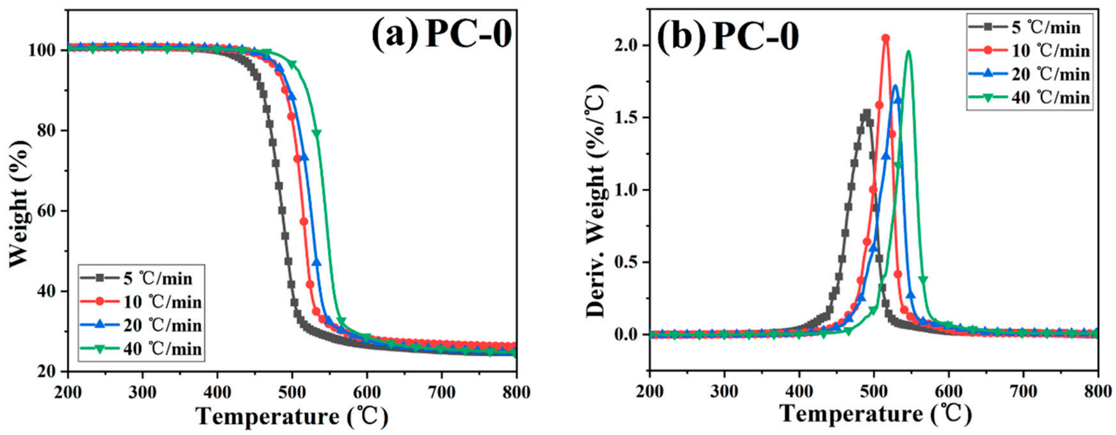

Figure S3 (a) TGA and (b) DTG curves of PC at different heating rates.

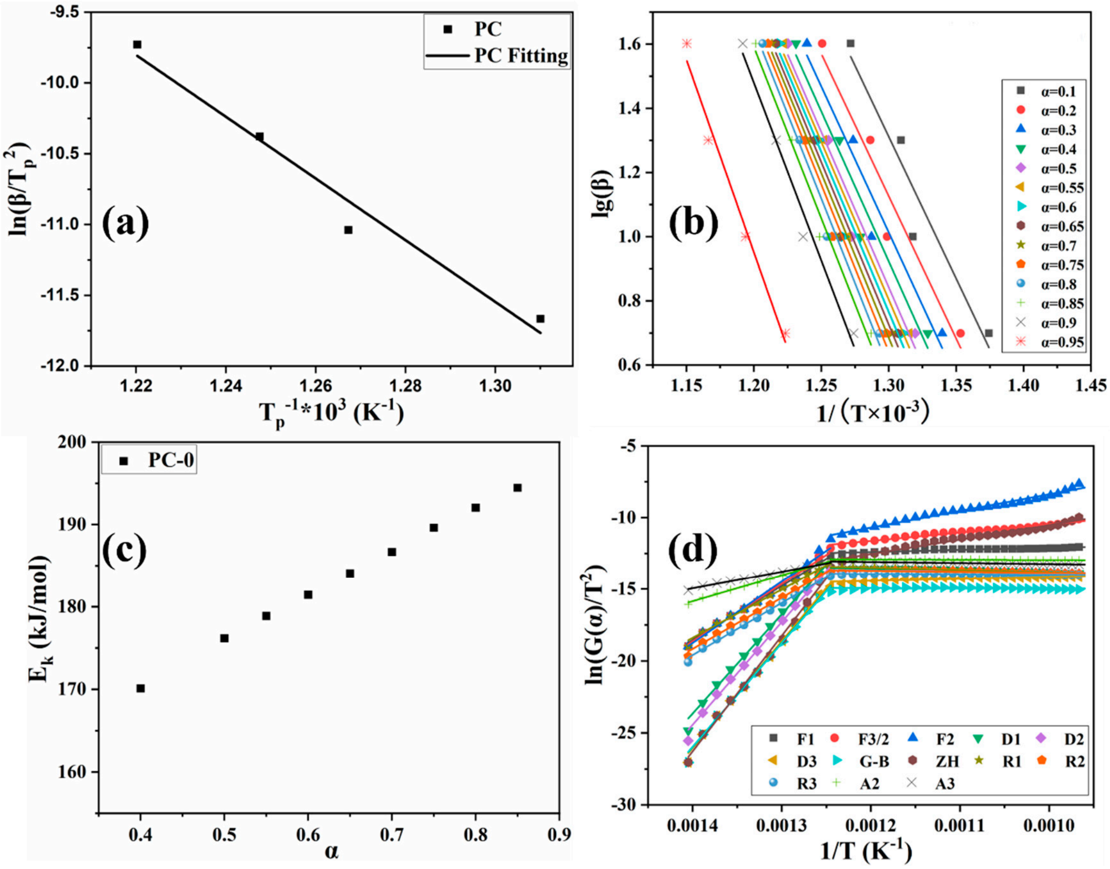

Figure S4 (a) Fitting curve of PC based on Kissinger method. (b) Fitting curve of PC based on F-W-O method. (c) Activation energy of PC at different conversion rates in F-W-O method. (d) Fitting curve of PC based on DAEM method.

Table S1 Fitting data of PC and its composite through Kissinger method.

Sample	β (℃ /min)	$1/T_p$ ($10^3 \cdot K^{-1}$)	$\ln(\beta/T_p^2)$	Slope	R’	E_k (kJ/mol)
PC-0	5	1.3100	-11.67	-21.80	-0.98574	181.27
	10	1.2673	-11.04			
	20	1.2475	-10.38			
	40	1.2203	-9.73			
PC-P0.3	5	1.3189	-11.653	-21.78	-0.99089	181.05
	10	1.2785	-11.022			
	20	1.2573	-10.362			
	40	1.2286	-9.7149			

Table S2 Fitting data of PC and its composite through F-W-O method.

α	PC-0		PC-P0.3	
	R	E_k (kJ/mol)	R	E_k (kJ/mol)
0.4	-0.97900	170.11	-0.99363	185.79
0.5	-0.98261	176.17	-0.99278	185.91
0.55	-0.98424	178.87	-0.99220	185.88
0.6	-0.98574	181.44	-0.99226	185.59
0.65	-0.98745	184.03	-0.99174	186.46
0.7	-0.98899	186.66	-0.99136	186.51
0.75	-0.99007	189.61	-0.99082	186.39
0.8	-0.99146	192.04	-0.99006	187.25
0.85	-0.99229	194.45	-0.98925	188.26

Table S3 Common kinetic models and typical mechanism functions.

Model		G (α)	f (α)	Reaction mechanism
Chemical reaction	F1	$-\ln(1-\alpha)$	$1-\alpha$	Grade 1 reaction
	F3/2	$2\left[(1-\alpha)^{-1/2}-1\right]$	$(1-\alpha)^{3/2}$	Grade 1.5 reaction
	F2	$(1-\alpha)^{-1}-1$	$(1-\alpha)^{2}$	Grade 2 reaction
Diffusion controlled reaction	D1	α^{2}	$1/2\alpha$	1D diffusion
	D2	$(1-\alpha)\ln(1-\alpha)+\alpha$	$-\ln(1-\alpha)^{-1}$	2D diffusion
	D3	$\left[1-(1-\alpha)^{1/3}\right]^{2}$	$1.5(1-\alpha)^{2/3}\left[1-(1-\alpha)^{1/3}\right]^{-1}$	3D diffusion
	G-B	$(1-2/3\alpha)-(1-\alpha)^{2/3}$	$1.5\left[(1-\alpha)^{-1/3}-1\right]^{-1}$	3D diffusion
	ZH	$\left[(1-\alpha)^{-1/3}-1\right]^{2}$	$1.5(1-\alpha)^{4/3}\left[(1-\alpha)^{-1/3}-1\right]^{-}$	3D diffusion
Phase boundary reaction	R1	α	1	1D reaction
	R2	$1-(1-\alpha)^{1/2}$	$2(1-\alpha)^{1/2}$	2D reaction
	R3	$1-(1-\alpha)^{1/3}$	$3(1-\alpha)^{2/3}$	3D reaction

Model		G (α)	f (α)	Reaction mechanism
Nucleation and growth	A2	$\left[-\ln(1-\alpha)\right]^{1/2}$	$2(1-\alpha)\left[-\ln(1-\alpha)\right]^{1/2}$	Stochastic kernelization
	A3	$\left[-\ln(1-\alpha)\right]^{1/3}$	$3(1-\alpha)\left[-\ln(1-\alpha)\right]^{2/3}$	Stochastic kernelization

Table S4 Correlation coefficient R' value obtained after fitting different pyrolysis mechanism functions of PC and its composites.

G(α)	PC-0 (435-530℃)		PC-P0.3 (435-526℃)	
	R'	E_k (kJ/mol)	R'	E_k (kJ/mol)
F1	-0.9984	314.3	-0.9926	339.8
F3/2	-0.9959	334.3	-0.9876	363.8
F2	-0.9913	356.3	-0.9801	390.8
D1	-0.9981	574.1	-0.9962	614.5
D2	-0.9989	593.9	-0.9959	636.8
D3	-0.9991	617.0	-0.9948	663.6
G-B	-0.9990	601.57	-0.9956	645.6
ZH	-0.9971	667.4	-0.9898	723.5
R1	-0.9980	280.8	-0.9961	301.1
R2	-0.9990	296.5	-0.9953	318.9
R3	-0.9990	302.2	-0.9946	325.6
A2	-0.9983	150.9	-0.9921	163.7
A3	-0.9982	96.39	-0.9915	104.9

Table S5 Mechanical performances of PC and its composites.

Samples	PC-0	PC-PTFE	PC-P0.1	PC-P0.3	PC-P0.5
Storage modulus (MPa) RT	2015	2145	2041	2046	2052
Tg (^{o}C)	157.2	157.5	156.6	155.9	155.1
Impact strength (kJ/m^2)	61.1	66.6	68.6	62.9	76.5
Tensile strength (MPa)	24.48	33.49	26.42	30.49	24.21
Elastic modulus (Pa)	1273.9	1259.9	1289.9	1224.2	1200.0

RT: Room temperature.
